# Five Years JAAOS Global Research & Reviews

**DOI:** 10.5435/JAAOSGlobal-D-22-00057

**Published:** 2022-04-05

**Authors:** Jeffrey S. Fischgrund


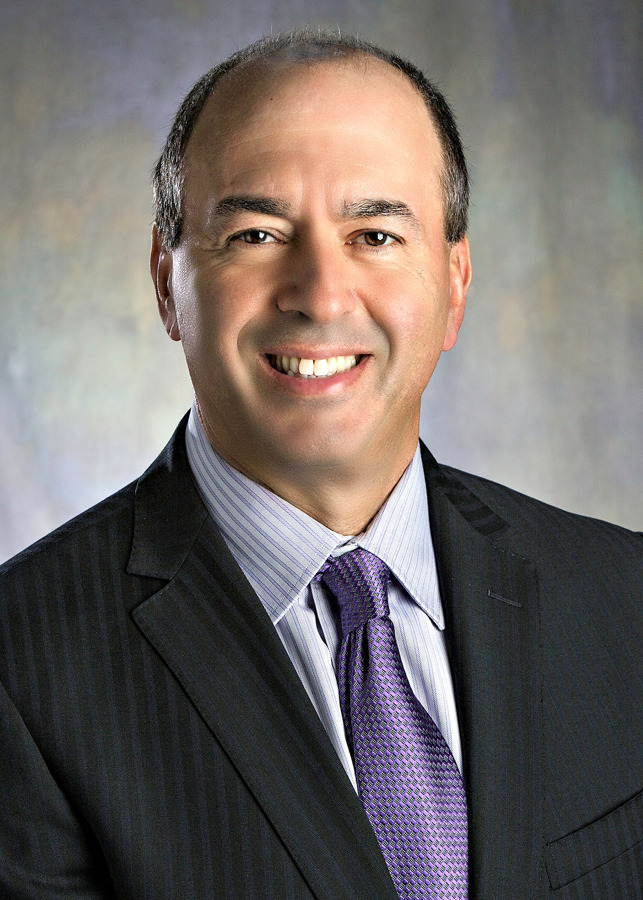


In 2017, the *Journal of the American Academy of Orthopaedic Surgeons* published the inaugural edition of the “Blue Journal”—*JAAOS Global Research & Reviews* (*JAAOS Global*). The mission of this *JAAOS* expansion was to improve the availability of quality orthopaedic knowledge through an open access, peer-reviewed, international journal. After 5 years of steady growth, I am proud to announce that we are achieving this lofty goal.

Since the publication of our first issue in March 2017, *JAAOS Global* has reached many critical milestones:

(1) Formation of an International Advisory Board, representing eight countries and five continents;
(2) Ten Deputy Editors from eight different countries;
(3) Indexed in MEDLINE, Scopus, PubMed Central, Emerging Sources Citation Index, and Directory of Open Access Journals;
(4) Over 90,000 unique visitors to the website;
(5) 366 submissions in 2021, an increase of 23% from 2020;
(6) Over 140 articles published in 2021 (30 published in 2017);
(7) Five *JAAOS Global* partnerships with international orthopaedic associations.

These accomplishments would not be possible without the assistance of our talented Deputy Editors and hundreds of reviewers (see list at https://links.lww.com/JG9/A211) from around the world. These volunteers provide an invaluable service to the Journal by using their expertise to provide constructive feedback to our authors. This has led to the early successes of *JAAOS Global* and positions the *JAAOS* portfolio for continued growth in the future.

Over the next few years, we will continue to expand our global partnerships. Our goal is to solicit more articles from international authors. This will help us to accomplish our mission of disseminating high-quality orthopaedic knowledge around the world.

